# Judicious use of low-dosage corticosteroids for non-severe COVID-19: A case report

**DOI:** 10.1515/med-2021-0250

**Published:** 2021-03-19

**Authors:** Jian Zhang, Zigang Tian, Lina Feng, Zhongming Yang, Bo Zou, Kun Li, Yingliang Zhang, Yaguo Wang, Joy Fleming, Wenyu Cui

**Affiliations:** Department of Infectious Diseases, Changchun Infectious Diseases Hospital, Changchun, Jilin, China 130123; Surgical Department of Tuberculosis, Changchun Infectious Diseases Hospital, Changchun, Jilin, China 130123; Internal Medicine Department of Tuberculosis, Changchun Infectious Diseases Hospital, Changchun, Jilin, China 130123; Department of Pleurisy, Changchun Infectious Diseases Hospital, Changchun, Jilin, China 130123; Key Laboratory of RNA Biology and CAS Center for Excellence in Biomacromolecules, Institute of Biophysics, Chinese Academy of Sciences, Beijing, China 100101; Changchun Infectious Diseases Hospital, Changchun, Jilin, China 130123

**Keywords:** SARS-CoV-2, cytokine storm, acute respiratory distress syndrome, methylprednisolone, personalized medicine

## Abstract

Inflammation-mediated lung injury in severe cases of infection with SARS-CoV-2, the aetiological agent of Coronavirus disease 2019 (COVID-19), can lead to respiratory failure and death, and therapies that block or ameliorate lung injury-associated inflammatory “cytokine storms” and progression to acute respiratory distress syndrome (ARDS) are urgently needed. Therapeutic use of corticosteroids for this purpose has been controversial because of conflicting reports on their efficacy and immunosuppressive behaviour. The WHO has strongly recommended treating critical COVID-19 patients with systemic corticosteroid therapy, but recommends against corticosteroid therapy in non-severe COVID-19 disease because of a lack of strong evidence on its efficacy. This retrospective case report describing the successful treatment of a non-severe COVID-19 case in Changchun, China, by judicious administration of corticosteroids using a personalized therapeutic approach was recorded to strengthen the evidence base showing how corticosteroid use in non-severe COVID-19 cases can be safe and efficacious. Alongside supportive care and lopinavir/ritonavir antiviral drugs, a low dosage of methylprednisolone was administered over a short period to attenuate lung inflammation. Regular chest CT scans guided dosage reduction in response to lesion absorption and improved lung condition. Judicious use of corticosteroids safely attenuated disease progression and facilitated rapid and complete recovery.

## 1 Introduction

The outbreak of COVID-19 that emerged in Wuhan, China, in December 2019, was declared a global pandemic by the WHO on 11 March 2020 [1]. To date (26 January 2021), there have been 99,363,697 confirmed cases worldwide and 2,135,959 associated deaths [2]. Clinical progression of COVID-19 generally begins with fever, fatigue, and a dry cough. The vast majority of cases are asymptomatic or resolve after only minimal symptoms, but in more severe cases, dyspnoea occurs about 1 week after disease onset and uncontrolled elevated levels of cytokine release, known as “cytokine storms,” can rapidly lead to disease progression, including acute respiratory distress syndrome (ARDS), septic shock, refractory metabolic acidosis, coagulation disorders, and ultimately death [3,4]. In the absence of standard effective treatments, therapy typically involves a combination of approaches, including administration of antiviral and immunomodulatory drugs, and treatments such as convalescent plasma, in addition to standard supportive patient care [5,6,7]. The development, optimization, and evaluation of more effective methods for the treatment of this acute respiratory infection are urgent.

Corticosteroid therapy has been used effectively to suppress the “cytokine storm” that contributes to the tissue injury associated with acute viral respiratory infections [8,9], but its use has been controversial because of its immunosuppressive action and other potential side-effects (hyperglycaemia, hypokalaemia, hypertension, gastrointestinal haemorrhage, avascular necrosis of bone, and nosocomial infections) [10,11,12,13]. Numerous clinical trials on the efficacy of corticosteroids in COVID-19 therapy have been initiated since the start of the COVID-19 pandemic. Positive results on the efficacy of dexamethasone for treating critically ill patients in the UK’s RECOVERY (Randomized Evaluation of COVID-19 Therapy) trial (involving 6,425 patients with severe COVID-19 randomized to 6 mg/day of dexamethasone or standard care) were reported in mid-June [14], halting enrolment in many other trials. Findings indicated that critically ill patients on ventilators receiving dexamethasone had a one-third lower risk of dying within 28 days than those who did not receive the drug, and the risk of dying was 20% lower in patients receiving oxygen therapy, but not on ventilators. Dexamethasone treatment, however, did not significantly alter 28-day mortality in patients receiving no respiratory support. A subsequent WHO prospective meta-analysis of data from seven clinical trials (including a total of 1,703 critically ill patients from five continents) supported the findings of the RECOVERY trial and concluded that efficacy could be attributed to corticosteroids as a group, not just dexamethasone [15]. The WHO issued two recommendations regarding corticosteroids in September 2020: a strong recommendation that systemic corticosteroids be used as a component of standard care for the treatment of patients with severe and critical COVID-19 (based on moderate certainty evidence), and a conditional recommendation based on low certainty evidence that corticosteroids not be used in the treatment of patients with non-severe COVID-19 because of the possibility of harm [16]. Further evidence of the efficacy of short-term low-dosage corticosteroid therapy in non-severe COVID-19 cases is sought, and indeed, some clinical trials have been completed and others are underway [17,18]. Here, to contribute to an evidence base on the safety and efficacy of corticosteroid therapy in non-severe COVID-19 cases, we provide a retrospective case report on the successful treatment of the index COVID-19 case in Changchun, Jilin province, China. Corticosteroid therapy was given to this moderate COVID-19 case using a personalized approach that involved regular monitoring and dosage adjustment according to treatment response. The patient experienced a rapid and complete recovery with no side-effects. Judicious use of corticosteroids in patients with non-severe infections and no underlying co-morbidities may significantly help to prevent disease progression.

## 2 Case report

**Figure 1:**
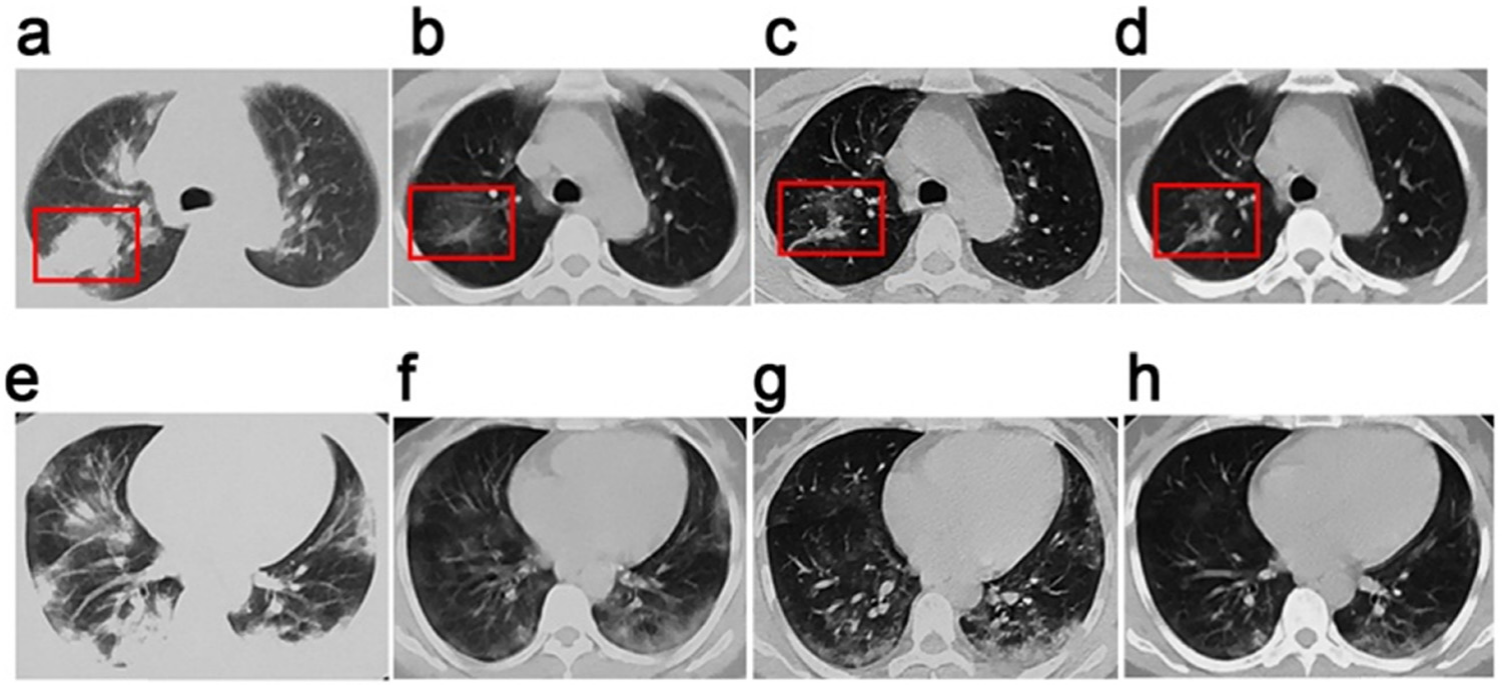
Chest CT images of a 41-year-old woman with SARS-CoV-2 infection. Chest CT images for a 41-year-old woman who presented at Changchun Infectious Diseases Hospital with fever and cough. (a–d) Images showing lesions in the posterior segment of the inferior lobe of the right lung. (a) Image taken at admission before the onset of treatment showing consolidated opacities in the right lung. (b and c) Images obtained on days 4 and 7 showing marked absorption of pulmonary consolidations. (d) Image taken on day 10 showing stripe-like opacities indicating recovery of the lung from infection. (e–h) Images of the lower lobe of the lung taken, (e), at the onset of treatment, (f), on day 4, (g), on day 7, and (h), on day 10, showing the infection and progressive treatment efficacy. Ground-glass opacities in the pulmonary pleural can be seen to reduce in the lower lobe of the lung from (e) to (h).

A 41-year-old woman travelled from Wuhan, Hubei province, China, to Changchun, Jilin province, on 19 January 2020. She attended the fever clinic of Changchun Infectious Diseases Hospital on the afternoon of 19 January, presenting with an intermittent fever (highest temperature 38.2°C) and cough, chest tightness, and fatigue that had persisted for 8 days. She was admitted to hospital the following afternoon after throat swab specimens collected from the upper respiratory tract tested positive for SARS-CoV-2 nucleic acid by RT-PCR (reverse-transcriptase polymerase chain reaction) [3,19]. She presented results of laboratory tests performed at Tongji Hospital in Wuhan on 17 January: lymphocyte count (low): 0.89 × 10^9^/L, neutrophils: 80.4%, serum CRP (C-reactive protein): 164.7 mg/L. Relevant multifunctional monitoring indicators on admission in our hospital were: transcutaneous oxygen saturation (SpO_2_): 93%, temperature: 37.4°C, respiratory rate: 20 breaths/min, pulse: 80 beats/min, blood pressure: 126/76 mm Hg. Auscultation of both lungs was unremarkable. Chest computed tomography (CT) showed consolidation in the right lung and peripheral ground-glass opacities in both lungs (Figure 1a and e). Her underlying health was good, and she had no known comorbidities. In line with relevant national guidelines in force at the time [20], she was diagnosed as a severe COVID-19 case and treated accordingly.

### 2.1 Treatment

#### 2.1.1 Phase I (days 1–4)

Moxifloxacin (0.4 g, IV, od) was used to prevent secondary infections, methylprednisone (40 mg, IV, od) was used to decrease inflammatory exudates in the lungs, acetylcysteine (0.2 g, po, tid) was given to prevent lung fibrosis, and lopinavir–ritonavir (2 × 200 mg/50 mg, po, tid) was used as an antiviral treatment. A chest CT scan performed on day 4 was compared to that taken at admission to assess treatment efficacy (Figure 1a, b, e, and f); evidence suggested absorption of pulmonary lesions in both lungs and of the inflammatory consolidation in the right lung. The patient’s temperature had returned to normal, and her cough, chest tightness, and fatigue were alleviated to some extent. Transcutaneous oxygen saturation (SpO_2_) increased to 97%. Laboratory tests were performed as required, beginning the morning after admission to hospital (HD2; Table 1).

**Table 1:** Clinical laboratory results for four points during hospitalization, from admission to discharge

Time from onset of symptoms	Day 9	Day 10	Day 16	Day 18
Time in hospital^a^	HD2	HD3	HD9	HD11
Blood parameter	21/1b	22/1	28/1	30/1
White blood cells (×10^9^/L)	8.9		11.4	12
Lymphocytes (×10^9^/L)	0.63		0.9	1.7
Lymphocytes (%)	7		8.2	14.3
Platelets (×10^9^/L)	362		466	446
Glucose (mmol/L)	4.5		5.41	5.19
Creatine (μmol/L)	32.6		46.4	43.05
Total bilirubin (μmol/L)	5.2		4.5	5.7
AST (U/L)	26		20	20
ALT (U/L)	37		31	27
LDH (U/L)	327		181	202
hs-Crp (mg/L)	14.3	13.7	3	1.32
PT (S)	11.2	10.9	12.6	13.4
APTT (S)	30.3	32.3	28.6	31.7
pH	7.42	7.35	7.28	7.38
PO_2_ mm Hg	90	94	95	95

a Number of days from admission to hospital.

b Calendar date.

#### 2.1.2 Phase II (days 5–7)

Lopinavir–ritonavir treatment was discontinued because of the improvements observed above, and the dosage of intravenous acetylcysteine was adjusted to 50%. Other elements of the treatment described above were maintained. Comparison of chest CTs taken at the end of Phases I and II indicated further improvement, with ongoing absorption of pulmonary lesions (Figure 1b, c, f, and g).

#### 2.1.3 Phase III (days 8–12)

Methylprednisone was replaced with prednisone because of the improvements observed, and the dosage was adjusted to 20 mg (IV, od). Comparison of chest CT scans from the end of phases II and III once again indicated steady absorption of pulmonary lesions in both lungs (Figure 1c, d, g, and h). By this point, the patient felt significantly recovered, and her cough, chest tightness, and fatigue were almost gone. Lymphocyte and CRP levels also returned to normal (Table 1). RT-PCR tests for SARS-CoV-2 nucleic acid were conducted on days 9 (27 January) and 11 (29 January) of hospitalization. Both tests returned negative results and the patient was discharged from hospital on completion of treatment on 30 January.

#### 2.1.4 Phase IV

The patient continued to take prednisone (10 mg, po, od) for a further 5 days after discharge to consolidate recovery. Telephone follow-up of the patient 10 days after discharge (and after 6 months for review) indicated that she was in good health and had not experienced any side-effects or complications.

This study was approved by the Changchun Infectious Diseases Hospital Ethics Committee (2020-CCID-TA-002) and the patient gave permission to publish this case report.

## 3 Discussion

The uncontrolled upregulation of cytokines, known as a “cytokine storm,” is a key factor determining the severity of SARS-CoV-2 infections and can lead to rapid progression to ARDS, increasing morbidity. SARS-CoV-2 infection causes a rapid immune response involving the activation of a range of host immune cells, including Th1, Th2, and Th17 helper cells, macrophages, dendritic cells, and neutrophils [21]. Hyperactivation of the immune response, however, results in abnormally high levels of inflammatory cytokines and chemokines [22], and the further recruitment of other immune cells leading to sustained release of cytokines and an aggressive inflammatory response which can have severe respiratory complications [23]. Targeted and timely therapeutic management of cytokine storm-induced lung inflammation is thus an attractive approach for attenuating disease progression and enhancing patient survival.

While corticosteroids are not the only drugs available for managing lung inflammation, they are widely available therapeutics that have a long history of clinical use and are effective in reducing levels of the inflammatory mediators involved in cytokine storms. Their binding to cytoplasmic corticosteroid receptors results in their translocation to the nucleus where they downregulate the activity of pro-inflammatory transcription factors such as NF-kB [24]. Corticosteroid receptor activation also regulates the transcription of anti-inflammatory genes [24]. However, their use in treating viral infections, including COVID-19, has been controversial [10] because of their immunosuppressive behaviour. On the one hand, the administration of corticosteroids in MERS (Middle East respiratory syndrome) patients, for example, was reported to delay viral clearance and increase plasma viral load if administered before viral replication was controlled [25]. On the other hand, their use in critically ill SARS (severe acute respiratory syndrome) patients was reported to reduce mortality [8,9]. While, in view of the inconclusive evidence, the WHO initially recommended against treating COVID-19 patients with corticosteroids [26], in the face of urgent clinical needs, an expert panel from the Chinese Thoracic Society published a consensus statement on the use of corticosteroids for treating COVID-19, recommending judicious short-term (≤7 days) use of low to moderate doses (≤0.5 mg/kg/day methylprednisolone or equivalent) for the treatment of seriously ill patients [27]. Treatment guidelines from the National Health Commission of China released in the early stages of the COVID-19 outbreak in China likewise recommended use of corticosteroid therapy (1–2 mg/kg/day) for severe and critical cases of COVID-19 [20]. As mentioned in the introduction, in view of the evidence emerging from recent clinical trials, the WHO now strongly recommends that short-term treatment with low-dosage systemic corticosteroids should be a component of standard care for critically-ill COVID-19 patients [16], but while there is no sufficiently strong evidence of benefit, patients with non-severe disease should not be treated with corticosteroids because of the possibility of harm. Studies on the use of corticosteroids in non-severe COVID-19 disease are ongoing; for example, emerging results from a US study suggest that an early short course of methylprednisolone in patients with moderate to severe COVID-19 can reduce the escalation of care required and improve clinical outcomes [17], and data from a study in France suggest that an early short course of corticosteroids combined with furosemide reduces the risk of invasive mechanical ventilation and 28-day mortality in non-severe COVID-19 patients [18].

In line with Chinese National Health Commission guidelines at the time [20], the patient in this case was considered as a borderline severe case (chest tightness, shortness of breath, SpO_2_: 93%, CT scan showing evidence of bilateral lung lesions and differences in density in the upper, middle, and lower lung regions), and systemic low-dosage corticosteroids were administered to address lung inflammation. Under WHO guidelines, however, this patient would have been considered as a moderate COVID-19 case (fever, cough, dyspnoea, SpO_2_: ≥90%), and the use of corticosteroid therapy would not have been recommended [7]. All evidence in this case, however, points to the efficacy of the judicious low dosage of corticosteroids administered over a short duration to assist recovery of the lungs from inflammation induced by SARS-CoV-2 infection, and for reducing pulmonary exudate accumulation. The initial dosage of corticosteroid (methylprednisolone) administered was calculated according to the body weight of the patient (52 kg); during treatment phases I and II (days 1–5), the dosage given, 40 mg/day (0.77 mg/kg/day), was low to minimize any side-effects of this treatment. While the use of corticosteroids is appropriate for treating lung inflammation in acute respiratory viral infections, we recognized that a judicious approach was required. Here, we achieved a marked reduction in corticosteroid use and lung inflammation, along with good absorption of pulmonary consolidations by administering a low dosage of corticosteroids over a relatively short duration. Evaluation of treatment progress based on comparisons of chest CT scans taken at the end of each phase of treatment allowed us to steadily reduce the initial dosage of corticosteroids, thus minimizing use. None of the complications associated with corticosteroid use were observed here during the treatment period or over the following 6 months after recovery. It is nonetheless important to monitor patients given this treatment in the short-medium term.

As mentioned above, a major concern with respect to the application of corticosteroid therapy is that, as an immunosuppressant, corticosteroids may delay viral clearance if administered before viral replication has been controlled [25]. In this case, however, the patient tested negative for SARS-CoV-2 nucleic acid on days 7 and 9 of treatment, indicating that corticosteroid use did not delay viral clearance. This is consistent with findings in other COVID-19 centres [28]. Indeed, it is possible that earlier testing might have indicated that this treatment contributed to viral clearance at an even earlier stage.

## 4 Conclusion

Our recommendation is that judicious and monitored use of low doses of corticosteroids in patients with moderate acute COVID-19 infections, when the absence of underlying co-morbidities does not indicate otherwise, may be sufficient to prevent moderate infections turning into severe/critical cases that develop ARDS and organ failure. Further studies are needed to confirm that the safety and efficacy of corticosteroid use observed in this case apply more widely to their use in non-severe COVID-19 cases in different populations, including the elderly.

## List of abbreviations

ARDS: acute respiratory distress syndrome
COVID-19: coronavirus disease 2019
CRP: C-reactive protein
CT: computed tomography
MERS: Middle East respiratory syndrome
RECOVERY: randomized evaluation of COVID-19 therapy
RT-PCR: reverse-transcriptase polymerase chain reaction
SARS: severe acute respiratory syndrome
SpO_2_: transcutaneous oxygen saturation
WHO: World Health Organization
